# Generation of a high yield vaccine backbone for influenza B virus in embryonated chicken eggs

**DOI:** 10.1038/s41541-023-00603-3

**Published:** 2023-02-10

**Authors:** Sadaf Aslam, Madhusudan Rajendran, Divya Kriti, Andrew Kurland, Jeffrey Johnson, Harm van Bakel, Florian Krammer, Adolfo García-Sastre, Juan Ayllon

**Affiliations:** 1 Department of Microbiology, Icahn School of Medicine at Mount Sinai, One Gustave L. Levy Place, New York, NY 10029 USA; 2 Global Health and Emerging Pathogens Institute, Icahn School of Medicine at Mount Sinai, One Gustave L. Levy Place, New York, NY 10029 USA; 3 Department of Genetics and Genomic Sciences, Icahn School of Medicine at Mount Sinai, One Gustave L. Levy Place, New York, NY 10029 USA; 4 Department of Pathology, Molecular and Cell-Based Medicine, Icahn School of Medicine at Mount Sinai, One Gustave L. Levy Place, New York, NY 10029 USA; 5 Department of Medicine, Division of Infectious Diseases, Icahn School of Medicine at Mount Sinai, One Gustave L. Levy Place, New York, NY 10029 USA; 6 The Tisch Cancer Institute, Icahn School of Medicine at Mount Sinai, One Gustave L. Levy Place, New York, NY 10029 USA; 7 Department of Health Sciences, University of Burgos, Burgos, Spain

**Keywords:** Influenza virus, Vaccines, Vaccines

## Abstract

Influenza B virus (IBV) strains are one of the components of seasonal influenza vaccines in both trivalent and quadrivalent formulations. The vast majority of these vaccines are produced in embryonated chickens’ eggs. While optimized backbones for vaccine production in eggs exist and are in use for influenza A viruses, no such backbones exist for IBVs, resulting in unpredictable production yields. To generate an optimal vaccine seed virus backbone, we have compiled a panel of 71 IBV strains from 1940 to present day, representing the known temporal and genetic variability of IBV circulating in humans. This panel contains strains from the B/Victoria/2/87-like lineage, B/Yamagata/16/88-like lineage and the ancestral lineage that preceded their split to provide a diverse set that would help to identify a suitable backbone which can be used in combination with hemagglutinin (HA) and neuraminidase (NA) glycoproteins from any IBV strain to be incorporated into the seasonal vaccine. We have characterized and ranked the growth profiles of the 71 IBV strains and the best performing strains were used for co-infection of eggs, followed by serial passaging to select for high-growth reassortant viruses. After serial passaging, we selected 10 clonal isolates based on their growth profiles assessed by hemagglutination and plaque-forming units. We then generated reverse genetics systems for the three clones that performed best in growth curves. The selected backbones were then used to generate different reassortant viruses with HA/NA combinations from high and low titer yielding wild type IBV. When the growth profiles of the recombinant reassortant viruses were tested, the low titer yielding HA/NA viruses with the selected backbones yielded higher titers similar to those from high titer yielding HA/NA combinations. The use of these IBV backbones with improved replication in eggs might increase yields for the influenza B virus components of seasonal influenza virus vaccines.

## Introduction

According to World Health Organization (WHO) the annual influenza epidemic results in about 1 billion infections, 3–5 million cases of severe illness and about 290,000-650,000 deaths^1^. Out of those, a significant number are due to influenza B virus (IBV) infections. As examples of the burden caused by these viruses, according to weekly influenza reports by the Centers for Disease Control and Prevention (CDC), roughly half of the deaths in the US during the 2019-2020 season, including most of the pediatric death reports, were due to IBVs^2^.

IBVs belong to the *Orthomyxoviridae* family and are segmented, single stranded, negative sense RNA viruses^3^. IBVs were first isolated in 1940 and primarily infect humans although they have been found in seals and pigs^4,5^. IBVs are known to cause epidemics, but its restriction to humans has reduced the chances of a IBV pandemic, since there are no animal reservoirs where antigenically unrelated IBV can reassort. In the 1980ies, two antigenically distinct lineages of IBVs based on the sequences of the hemagglutinin (HA) segment of the virus were identified and in 2002 co-circulation of the two different lineages was detected. The two lineages are denoted as B/Yamagata/16/88-like (Y) and B/Victoria/2/87-like (V) viruses, or simply *Victoria*- and *Yamagata*-like, based on their prototype ancestral strains^6^.

In the last 20 years, there has been some progress in the development of next generation seasonal influenza vaccines. There are several manufacturing platforms that are currently being used, such as cell-based, protein-based, and egg-based. For influenza A virus, cell-based and egg-based seasonal vaccines utilize the internal segments of a high yield laboratory strain as backbone, in combination with HA and NA of the recommended strain for the season. Historically, re-assorted influenza virus vaccine seeds were made by co-infection of the selected strain with the laboratory strain, in the presence of antibodies against the HA and NA of the laboratory strains, to generate re-assorted viruses that had the internal segments of the laboratory strains conferring high-growth properties and the HA and NA of the desired vaccine strain^7^. However, due to the advancement in reverse genetic techniques, influenza virus vaccine seeds can now be generated by transfecting plasmids that contain internal segments of the high growth laboratory strains, and plasmids that express HA and NA of the desired circulating strains. This allows for faster generation of reassortant viruses for vaccine production^8–10^. However, this technology is not broadly used across all manufacturers. Many companies still use the co-infection of laboratory and strain with desired glycoproteins in the presence of antibodies to generate reassortants.

The current influenza manufacturing process takes approximately 7 months from strain selection to availability of enough vaccine doses^11,12^. Production-wise, it is of critical importance to have a high titer yielding backbone strain to reach the required production goals. The preferred backbone for influenza A virus vaccines is A/PR/8/34^7^, while there is no specific high titer yielding backbone donor strain for IBV. Historically, various strains have been used as the high titer yielding backbone strains for IBV, such as, B/Lee/40, and many times the wild type strain has been used without reassortment to generate the vaccine seed. There have been several attempts made to generate a high titer yielding seed virus for IBV, through reassortments, and by introducing several growth enhancing mutations into the six internal segments of a selected strain^13^. However, this method generated two separate backbones for the two main lineages of IBVs currently circulating, instead of one single backbone that can be used for all IBVs^13^.

In this study, our main goal was to generate a backbone that can be used to generate recombinant viruses that yield high titers for any IBV strain, regardless of the lineage of the wild type strain, in the most used manufacturing substrate for influenza vaccines, embryonated chicken eggs. We achieved this by testing several strains and performing competitive reassortment assays to select the desired backbone. Finally, reverse genetics was used to establish various recombinant viruses to test whether the selected internal virus gene backbone for IBV yielded high growth vaccine viruses when reassorted with the HA and NA genes of both high and low titer yielding wild type IBV strains.

## Results

### Standard conditions and testing a panel of influenza B virus strains

**Fig. 1:**
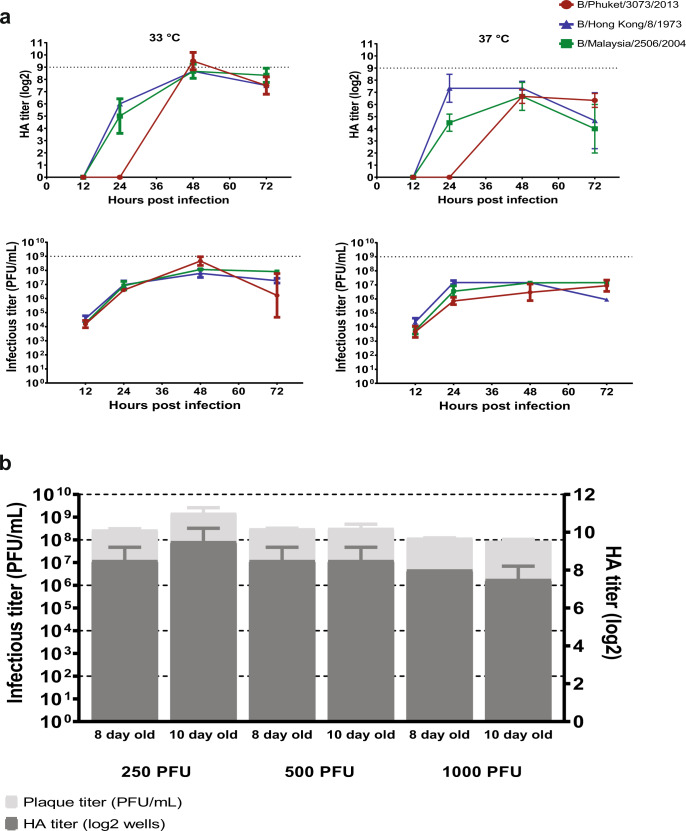
Optimal growth conditions for growing IBV in eggs. **a** Embryonated chicken eggs were injected with three representative strains to test for optimal temperature and optimal time post infection for growing IBV in triplicates and titers were determined by HA and plaque assays. Viruses were harvested 12, 24, 48 and 72 h post infection and eggs were incubated at 33 or 37 °C. **b** One representative strain (B/Malaysia/2506/2004 MA) was used to determine optimal egg age and inoculum for growing IBV in duplicates, and titers were determined by HA and plaque assay. 8 and 10 day old embryonated chicken eggs were used, and three different inoculums were tested. Dotted lines represent reference to the different titers. Two-way ANOVA with Tukey’s multiple comparisons test was performed to compare mean differences between different inoculums and egg ages. Statistical significance was considered when *p* ≤ 0.05 and marked on the graph.

First, we set out to determine the best possible growth conditions for IBV strains in embryonated chicken eggs, so we could use them later for the selection of the optimized backbones. We started testing for optimal temperature, and time post infection, using three representative model strains of B/Phuket/3073/2013 (B/Yamagata/16/88-like), B/Malaysia/2506/2004 (B/Victoria/2/87-like), and B/Hong Kong/8/73 (ancestral). We tested two different temperatures and several time points post-infection. The three strains grew to the highest infectious titers and hemagglutination (HA) titers at 33 °C and 48 h post-infection (Fig. 1a), independently of their lineage. Thus, from there onward we continued using B/Malaysia/2506/2004 MA (mouse adapted) as an internal control and as a reference strain. Then we determined starting infectious dosage and egg age by testing three different inocula (250, 500 and 1000 PFU (plaque forming unit)) and two different egg ages (8 and 10 days old) at 33 °C and 48 h post infection. The difference in titer was not statistically significant but there was a trend that B/Malaysia/2506/2004 MA had the highest HA and plaque titers in 10-day old eggs, with the 250 PFU inoculum (Fig. 1b).

**Fig. 2:**
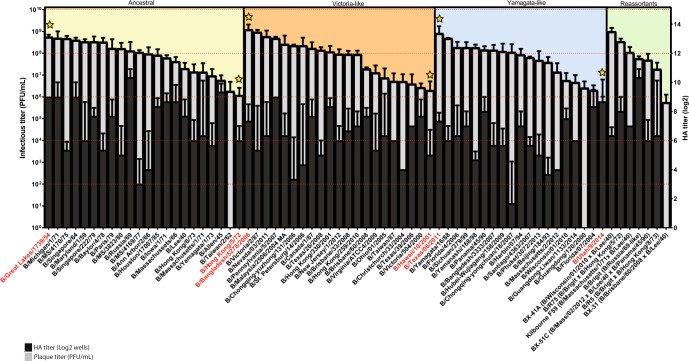
Peak titers for 71 different IBV strains in embryonated chicken eggs. A panel of IBV strains were tested in optimal conditions (10 day old embryonated chicken eggs, 33 °C, 48 h time post infection, and 250 PFU inoculum) in triplicates. The panel contained strains from ancestral linage, B/Victoria-like, B/Yamagata-like, and reassortant viruses. HA and plaque assay titers are shown (black and gray bars respectively), the strains are ranked from highest to lowest plaque titers within each lineage type. Plaque titers for all the strains ranged from 10^5^ to 10^9^ PFU/mL (Plaque forming unit (PFU)) (*n* = 3, error bars represent standard deviation). (Strains highlighted in red and marked with stars indicate the low and high titer yielding strains later tested in combination with optimal backbones).

**Fig. 3:**
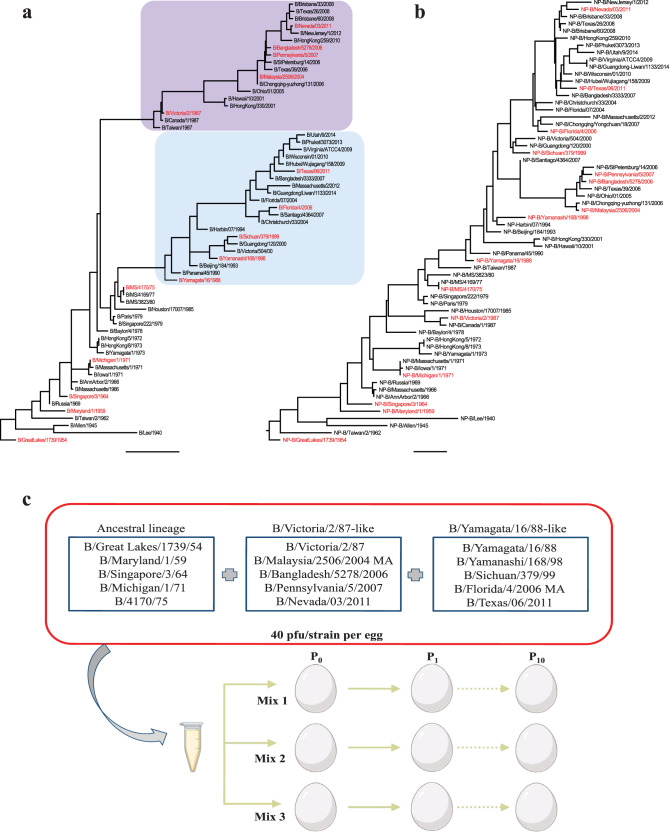
Phylogenetic analysis of nucleoprotein and hemagglutinin segments from the 71 IBV strains and schematic description of co-infection experiment. Nucleotide sequences for **a** HA (scale bar: 0.02) and (**b**) NP (scale bar: 0.007) were aligned using Clustal Omega and trees were visualized using Figtree. Highlighted in red are the 15 strains used for coinfection. (Scale bar indicates the percentage of change in nucleotide). **c** 15 highest growing IBV strains based on plaque titers were mixed at a constant concentration of 40 PFU/strain and 200 μl of the mixture was injected into three different eggs (labeled: Mix 1, Mix 2, and Mix 3). The virus was serial passaged ten times in eggs (10-day old eggs). In between passages, the virus was diluted to 10^-5^ and 200 μl was injected into the eggs. Virus titer was monitored by HA and plaque assay during the passages.

We then fixed the growth conditions as 33°C, 48 h post infection, 10 day old eggs, and 250 PFU inoculum, and proceeded to use them to assess the peak growth titers of 71 IBV strains (Fig. 2), which has been described before^14^. The phylogenetic analysis of the HA and nucleoprotein (NP) segments (Fig. 3a, b) of all the viruses used in this collection shows the broad distribution and temporal diversity of this panel of IBV strains.

### Co-infection and reassortant generation

Next, we selected 15 strains that had high infectious titers in eggs, from the panel of 71 strains. Specifically, we selected five strains with the highest titers from each group B/Yamagata/16/88-like, B/Victoria/2/87-like, and ancestral for co-infection. For B/Florida/4/2006, we used the MA version since the reverse genetics system was already established and available. The selected strains are highlighted in red in the two phylogenetic trees shown in Fig. 3a, b. We pooled all the strains at a constant titer of 40 PFU/strain (total volume: 200 µl/egg) and infected three 10-day old eggs labeled Mix 1, Mix 2, and Mix 3, respectively. These samples were serially passaged in eggs for 10 passages. Between each passage, collected allantoic fluid was diluted to 10−^5^ (by volume) and 200 µl were injected into fresh eggs (Fig. 3c). We monitored hemagglutination and plaque titers during the passages. Throughout the passages viral titers were relatively high, ranging between 10^7^ to 10^8^ PFU/mL. To assess the genetic diversity of the passaged mixtures, after passage 10, RNA-seq was done on all three final samples, indicating polyclonal populations in all of them. Polyclonal populations were defined by the detection of multiple single nucleotide variants.

### Clonal isolates

**Fig. 4:**
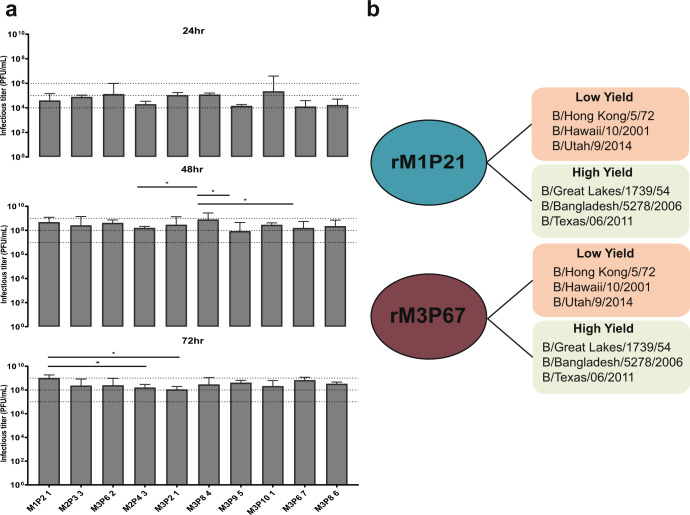
Growth kinetics of clonal isolates and list of recombinant viruses generated. **a** Growth kinetics of 10 clonal isolates was performed in eggs in triplicates and plaque titers were measured at 24, 48, and 72 h post infection in 10 day old embryonated chicken eggs. **b** Two of the highest growing clonal isolates were selected to generate reassortant viruses (M1P21 and M3P67) with HA/NA of low and high titer yielding viruses. (*n* = 3, error bars represent standard deviation). Two-way ANOVA with Tukey’s multiple comparisons test was performed to compare mean differences between different clonal isolates at different time points. Statistical significance was considered when *p* ≤ 0.05 (∗*p* < 0.05, ∗∗*p* < 0.01, ∗∗∗*p* < 0.001, ∗∗∗∗*p* < 0.0001); NS not significant and marked on the graph.

In order to filter the large genetic diversity and polyclonal populations assessed after the last passage, we picked multiple plaques from each of the three samples (a total of 19 plaques) and amplified them in eggs. All 19 clones were deep sequenced and analyzed. Since some of the plaque purified viruses still had a mixed population for some segments, we repeated the process by selecting 5 clones per initial plaque and amplified them in eggs for a total of 95 clonal isolates. After analyzing the data, we found 31 clonal isolates that did not have multiple single nucleotide variants. The identities of each segment were identified by aligning the consensus sequences from RNA-seq with the consensus sequences of each of the fifteen strains used for co-infection. We labeled each segment for the strain that aligned closest to the original sequence (Table 1 and supplementary table 3). We selected top 10 clonal isolates out of the 31 clonal isolates based on their hemagglutinin and plaque titers (a combination of PFU/HAU ratio and PFU titers) (Supplementary table 1). We performed growth kinetics in eggs with the selected 10 clonal isolates to determine the top three clonal isolates that grew to high titers (Fig. 4a). The three clonal isolates selected for establishing reverse genetics were M1P21, M3P84 and M3P67.

**Table 1:** Genetic characteristics of the top ten clonal isolates.

**M1P2 1**			**M2P3 3**	
PB1	Penn/07	K741R	Michigan/71	
PB2	Penn/07		Yamagata/88	
PA	Texas/11	K489E, V553M	Texas/11	K489E
HA	Florida/06 (MA)		Florida/06 (MA)	
NP	Michigan/71		Michigan/71	
NA	Great Lakes/54		Michigan/71	
M	Texas/11		Texas/11	
NS	Malaysia/04 (MA)		Malaysia/04 (MA)	
**M3P6 2**			**M2P4 3**	
PB1	Penn/07		Penn/07	
PB2	Penn/07		Penn/07	
PA	Texas/11	K489E	Texas/11	K489E
HA	Florida/06 (MA)		Texas/11	
NP	Michigan/71		Michigan/71	
NA	Yamagata/88	P42S, T44I (NB)	Michigan/71	
M	Texas/11		Malaysia/04 (MA)	
NS	Florida/06 (MA)	K70E (NS1)	Malaysia/04 (MA)	
**M3P2 1**			**M3P8 4**	
PB1	Penn/07		Penn/07	
PB2	Yamagata/88		Yamagata/88	
PA	Texas/11	K489E	Texas/11	K489E
HA	Florida/06 (MA)		Florida/06 (MA)	
NP	Michigan/71		Texas/11	
NA	Michigan/71		Michigan/71	
M	Texas/11		Texas/11	
NS	Penn/07		Penn/07	
**M3P9 5**			**M3P10 1**	
PB1	Penn/07		Penn/07	
PB2	Yamagata/88		Bangladesh/06	
PA	Texas/11	K489E	Texas/11	K489E
HA	Florida/06 (MA)		Florida/06 (MA)	
NP	Texas/11		Yamagata/88	E535G
NA	Michigan/71		Michigan/71	
M	Florida/06 (MA)		Texas/11	
NS	Penn/07		Malaysia/04 (MA)	
**M3P6 7**			**M3P8 6**	
PB1	Penn/07		Penn/07	
PB2	Penn/07		Yamagata/88	G632R
PA	Texas/11	K489E	Texas/11	K489E
HA	Florida/06 (MA)		Florida/06 (MA)	
NP	Michigan/71		Texas/11	
NA	Yamagata/88	P42S, T44I (NB)	Michigan/71	
M	Texas/11		Texas/11	
NS	Penn/07		Penn/07	

Nucleotide sequences of each segment from the clonal isolates were aligned against the nucleotide sequence of the wild type strains used for co-infection to identify the closest match. The respective amino acid changes are listed. ((NB) indicates mutations in the NB ORF on segment 5/NA, (MA) indicates mouse adapted, and (NS1) indicates mutations in the NS1 ORF on segment 8/NS). (Strain names: B/Florida/4/2006 MA, B/Great Lakes/1739/54, B/Malaysia/2506/2004 MA, B/Michigan/1/71, B/Pennsylvania/5/2007, B/Texas/06/2011, B/Yamagata/16/88, B/Bangladesh/5278/2006).

### Reassortant viruses

We then generated complete reverse genetics systems for all three selected high growth viruses. We cloned all 8 segments of M1P21, M3P84 and M3P67 into pDZ vectors^15^ (Fig. 4b). These 8 plasmids were transfected into 293 T cells and recombinant viruses were generated as described before^16^. Next, we selected low titer yielding strains from B/Yamagata/16/88-like, B/Victoria/2/87-like and ancestral groups based on the peak titers from the initial 71 IBV strain panel (Fig. 2, marked with a star and highlighted in red) and cloned their HA and NA segments into pDZ vectors. We used B/Hong Kong/5/72 (ancestral), B/Hawaii/10/2001 (B/Victoria/2/87-like) and B/Utah/9/2014 (B/Yamagata/16/88-like) as the low titer yielding viruses.

We also tested HAs and NAs of high titer yielding viruses to see if we can maintain or increase their titer even further with our backbone constructs. We used B/Great Lakes/1739/54 (ancestral), B/Bangladesh/5278/2006 (B/Victoria/2/87-like) and B/Texas/06/2011 (B/Yamagata/16/88-like) as the high titer yielding viruses (Fig. 2, marked with a star and highlighted in red). We generated recombinant M1P21 and M3P67 viruses and rescued them with different combinations of HA and NA segments from low and high titer yielding strains. We did not test the M3P84 backbone because this virus did not rescue very efficiently with the different combinations. However, we were able to rescue different combinations of viruses with M1P21 and M3P67 backbones in the first attempt.

### Growth kinetics using recombinant viruses

**Fig. 5:**
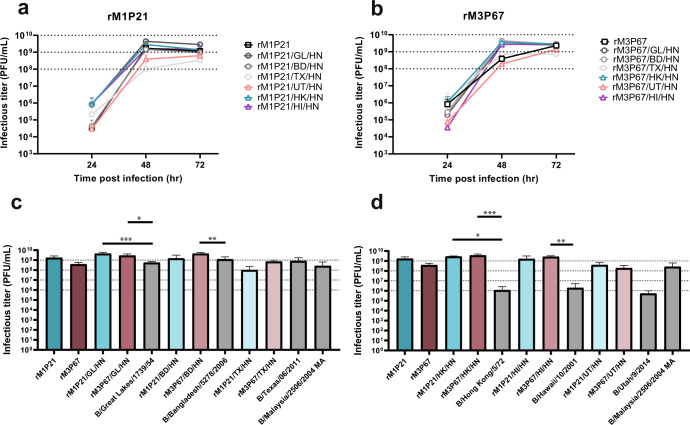
Growth kinetics and peak titers of recombinant viruses with different combination of HA/NAs. Growth kinetics of recombinant viruses with HA/NA of low and high titer yielding viruses with **a** M1P21 and (**b**) M3P67 backbones in eggs were performed in triplicates and plaque titers were measured at 24, 48 and 72 h post infection in 10 day old embryonated chicken eggs. Peak titers at 48 h were compared between (**c**) high titer yielding WT strains and recombinant viruses with high titer yielding HA/NA with the two backbones and (**d**) low titer yielding WT strains and recombinant viruses with low titer yielding HA/NA. (*n* = 3, error bars represent standard deviation) One-way ANOVA with Tukey’s multiple comparisons test was performed to compare mean differences between recombinant viruses and the respective WT strains. Statistical significance was considered when *p* ≤ 0.05 (∗*p* < 0.05, ∗∗*p* < 0.01, ∗∗∗*p* < 0.001, ∗∗∗∗*p* < 0.0001); NS not significant and marked on the graph.

To validate if the high titer yielding backbone indeed increases the titer of reassortants, we performed growth kinetics using the 14 recombinant viruses that we generated (Figs. 5a, b). We conclude that the titers for recombinant viruses with HA/NA derived from high titer yielding strains remained high, while the recombinant viruses with HA/NA from low titer yielding strains were higher than those of the corresponding wild type strains. A comparison of the peak titers of the recombinant viruses with the wild type strains at 48 h post infection is depicted in Figs. 5c, d. There was a 100-fold increase (10^6 to 10^8 PFU/mL or higher) in plaque titers when the HA/NA of low titer yielding viruses was reasserted with the backbone of the high growth selected viruses (Fig. 5d). And the titers for recombinant viruses with HA/NA derived from high titer yielding strains were still high and similar to the original titers (Fig. 5c). We observed statistical significance for difference in titers for some of the reassortant viruses versus WT strains. This indicates that these IBV backbones are efficient at generating reassortant viruses and can increase the titer for low titer yielding viruses regardless of the lineage of the virus.

### HA levels of high-growth reassortants

**Fig. 6:**
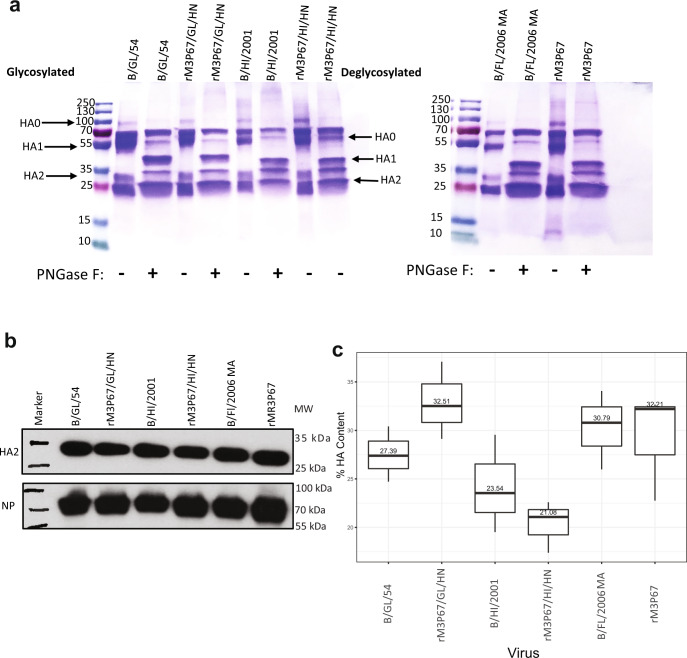
Characterization of HA content in the reassortant and WT viruses. Wild type and recombinant viruses were grown in 10 days old embryonated eggs and concentrated using 30% sucrose cushion. **a** 20 µg (total protein) of purified virus was treated with PNGase F. 10 µg (total protein) was loaded in 12% SDS-PAGE gel and stained with coomassie. Treated and untreated samples were run to see the shift in mobility. **b** 20 ng (total protein) of purified virus was denatured by heating and run on a 10% reducing denaturing SDS-PAGE and immunoblotted with antibody against HA (KL-BHA-4C10) for HA2 and NP (polyclonal commercial) served as a loading control. (MW = molecular weight, kDa = kilodalton). **c** Boxplot showing percent of HA content in total protein. Samples were digested with trypsin and spiked with heavy HA peptide (QLPNLLR or SKPYYTGEHAK) (*n* = 3, independent experiments). The amount of HA using each peptide is averaged to calculate HA content. The box plots are in the Tukey style, central line indicates median, the hinges of the box indicate the 25^th^ and 75^th^ quartiles of data. The whiskers indicate the largest/smallest values no further than 1.5 * IQR from the closest hinge (IQR inter-quartile range). Viruses used in all the experiments were the following B/Great Lakes/1739/54, B/Hawaii/10/2001, B/Florida/4/2006 MA (control), rM3P67 (control), rM3P67/GL/HN, rM3P67/HI/HN.

Finally, we wanted to determine the HA protein levels expressed and incorporated by the recombinant high growth viruses, as HA amount is the main factor used for standardizing vaccine batches. For this we used rM3P67/GL/HN, rM3P67/HI/HN, rM3P67 and the corresponding wild type strains. As a control for HA in rM3P67 we used B/Florida/4/2006 MA WT virus. First, we purified the recombinant viruses and wild type viruses using 30% sucrose cushion. We treated 20 µg of total protein with PNGase F and ran 10 µg of the treated sample on a 12% reducing denaturing sodium dodecyl sulfate polyacrylamide gel electrophoresis (SDS-PAGE) and stained with Coomassie blue (Fig. 6a). This showed that the virus preparations were pure, as indicated by the absence of smear, and the overall total protein pattern for reassortant and WT strains was same regardless of treatment. Next, we looked at HA protein only. We ran equal amounts of total protein (20 ng) for all the viruses in 10% SDS-PAGE gel and immunoblotted by probing with anti-HA mAb KL-BHA-4C10^17^. We compared HA2 levels across all the viruses and saw that the HA2 levels were similar across the recombinant and wild type viruses (Fig. 6b). A commercial polyclonal antibody against NP was used as a loading control and NP levels were similar across the viruses as well. Lastly, we quantified total protein concentration using Bradford assay (Supplementary table 2) for mass spectrometry based quantification of HA peptide. We digested 50 µg (total protein) of each concentrated virus with trypsin and spiked it with two different heavy peptides (individually). Each of the sample was analyzed on Orbitrap Eclipse mass spectrometry system. Percent of HA content in total protein was calculated by averaging the amount of HA obtained from the two peptides (Fig. 6c). Precursor chromatograms for each peptide and virus replicate showing precursor intensity and retention time (Supplementary fig. 1, 2) were used to calculate HA concentration. We observed slightly higher amount of HA concentration in recombinant reassortant viruses in comparison to the wild type strains from which the HA derives, which in combination to the high titers that can be obtained from them lead to a high yield production system for otherwise difficult to grow IBV vaccine strains.

## Discussion

The ongoing coronavirus disease 2019 (COVID-19) pandemic has highlighted the critical relevance of vaccines in combating currently circulating and newly emerging infectious diseases. The outstanding speed at which these vaccines have been mass-produced has also shown how relevant it is, not only to develop protective immunization strategies, but also the means by which they can be deployed in the most efficient manner.

Currently available influenza vaccines are suboptimal and need to be reassessed and reformulated every year, leading to a lengthy production time of 7 months, assuming the process goes smoothly, and vaccine strains are generated efficiently. Unfortunately, this is not always the case, as the yields obtained may vary greatly among different strains. While this can be circumvented for IAV by using well characterized, optimized backbones^18,19^, no such systems are currently in use for IBV and too often wild type IBV strains have to be used directly as vaccine seeds.

In this study, we generated two IBV vaccine backbones that can be used in combination with HA/NA from the two IBV lineages, leading to high titers for vaccine production in eggs. We generated these backbones by reassorting and performing competitive reassortment experiments among 15 high growing IBV strains, and screening 91 clonal isolates for high growth after two rounds of plaque purification. Out of the 91 clonal isolates we selected 3 clonal high growth isolates to establish reverse genetic systems. We generated recombinant reassortant viruses with HA/NA derived from representative low and high titer yielding IBV strains from B/Yamagata/16/88-like lineage, B/Victoria/2/87-like lineage and ancestral lineage. These reassortants maintained the high titer in eggs of the viruses that were already growing to high titers and also increased the titers of low titer yielding strains by 100-fold (10^6 to 10^8 PFU/mL or higher). Furthermore, we analyzed the HA content of the different viral preparations and found that the amount per viral particle in our recombinant optimized backbones is comparable to that on the wild type virions.

During sequence alignment and analysis, we identified mutations (Table 1 and supplementary table 3) that were present in various segments. Some of the amino acid changes were in the surface glycoprotein, NA. We also identified a mutation (P42S) common for B/Victoria/2/87-like^20^ viruses in one of the NAs that belongs to B/Yamagata/16/88-like virus in our alignment. We also found interesting amino acid changes in some of the internal segments. In clonal isolate M3P6 2, we identified an amino acid change at position 70 in NS1 protein. The mutation is K70E and it has been known as a site for SUMOylation in NS1 of IAV^21^. We suggest that this mutation plays a similar role in IBV, but further experiments would need to be performed to confirm this. We have also identified various mutations in PB1, PB2, PA and NP proteins and we would like to study them in detail to identify which mutations play a role in increasing the titer. Specifically, of the final two selected backbones, M1P21 has a K741R mutation in PB1, and both M1P21 and M3P67 share a K489E mutation in PA. It will be interesting to find out whether these mutations contribute to their high-growth properties and if they prevent K modification by ubiquitination, ISGylation and/or SUMOylation. We observed few changes in the non-coding regions. The relevance of the mutation (deletion of A at nucleotide 45) in NS of B/Malaysia/04 is not likely to significantly impact viral fitness, as it appears in both high and not-high growing clones. Other mutations such as addition of AA at nucleotide 47-48 in NP of B/Michigan/71 from clone M2P4 3 and nucleotide changes at position 10 in segments PB1 (B/Michigan/71 (A- > G)) and PB2 (B/Yamagata/88 (G- > A)) could be playing a role in polymerase binding, according to previously reported data^22^ and therefore may be an interesting subject for future studies.

Based on our findings, we suggest that these backbones could be used as vaccine backbones for IBV strains to be included in seasonal influenza virus vaccines. Although we did observe high-growth properties for all six representative HA/NA combinations for both backbones, the identification of two independent backbones might help in case one of the backbones is not optimal for a specific HA/NA combination.

Our work has been focused on the embryonated egg vaccine production and as such has been optimized for this platform. Embryonated eggs are a fast, reliable and cost-effective system that greatly expands vaccine production capabilities to a broader range of countries^23,24^ and so remain a critical technology for global influenza vaccination. It may not be only relevant for global influenza vaccination but any strategies that involve eggs for vaccine production, for example, some vaccines against SARS-CoV-2 are being produced in eggs^24^. Additionally, for other platforms competitive reassortment strategy could be used to select IBV backbones for vaccine production in other systems, such as tissue culture. And this strategy could also be used for generating backbones for other virus types for vaccine production.

## Methods

Institutional approval was not needed for any of the experiments performed in this paper.

### Cell culture

Madin–Darby canine kidney (MDCK) epithelial cells and human embryonic kidney 293 T cells were cultured in Dulbecco’s modified Eagle’s medium (Gibco) supplemented with 10% fetal bovine serum (HyClone) and 10 U penicillin per mL, 10 mg streptomycin per mL (Gibco) and grown at 37 °C and 5% CO_2_.

### Virus growth in eggs, plaque assay, and hemagglutination assay

IBV strains were grown in 8–10 day old embryonated chicken eggs (Charles River) for various time points and temperatures. Allantoic fluid was harvested and spun at 290 x g for 5 min at 4 °C and supernatant was aliquoted and frozen at −80 °C. Viral titer was determined using standard plaque assay on MDCK cells and immunostained using pool of monoclonal mouse antibodies against various strains of IBV. For plaque purification, individual plaques were identified and collected using 200 μl pipets and mixed with phosphate buffered saline (PBS) (Gibco) and injected into eggs for amplification. Hemagglutination assay^14^ was performed by serially diluting (1:2) virus supernatant with PBS (Gibco) in a V-bottom 96-well plate (Fisher) and 50 µl of 0.5% chicken red blood cells were added and incubated at 4 °C for 30 min. Hemagglutination units were determined by the reciprocal of the last dilution which showed complete hemagglutination.

### Cloning influenza B virus segments into rescue plasmids

Viral RNA was extracted from the allantoic supernatant using E. Z. N. A. Viral RNA kit (Omega Bio-Tek) according to the manufacturer guidelines. Viral segments were amplified by RT-PCR using Superscript III one-step RT-PCR kit (Invitrogen) and segment specific primers (supplementary table 5). RT-PCR products were gel purified (QIAGEN). pDZ plasmid was digested with *Sap*I restriction enzyme (New England biolabs) and treated with alkaline phosphatase (New England biolabs) and gel purified. Purified RT-PCR products were cloned into digested pDZ plasmid using In-Fusion kit (Takara bio). Plasmid sequences were confirmed by Sanger sequencing (Psomogen).

### Generation of recombinant influenza B viruses

Recombinant IBVs were generated by transfection of 293 T cells with plasmids containing viral segments^16^. Briefly, 8 plasmids encoding viral segments are co-transfected using TransIT LT1 (Mirus) and optimem (Gibco) according to manufacturer guidelines. 293 T cells (2 × 10^5^ cells/mL) are mixed with DNA/TransIT LT1 mixture and plated in a 6-well plate. 24 h later media is changed to infection media (1XMEM (Gibco)), 1% penicillin/streptomycin, 0.2% BSA (MP biomedicals), 1 mg/mL of N-tosyl-L-phenylalanine chloromethyl ketone (TPCK)-treated trypsin and the cells are incubated for 2 days at 33 °C. The supernatant is harvested and injected into 8–10 day old embryonated chicken eggs and incubated for 3 days at 33 °C. Allantoic fluid is harvested and hemagglutination assay is performed to check for presence of virus. Viruses were plaque purified and stocks were aliquoted and stored at −80 °C.

### Deep sequencing and data analysis

In order to get consensus sequences and monitor polymorphisms during passage, viruses were deep sequenced. Viral RNA was extracted and prepared for RNA-seq^25^. Briefly, viral RNA was extracted using E. Z. N. A. viral RNA kit (Omega Bio-Tek) and the samples were prepared to run on illumna. Raw data was aligned using NGen DNASTAR lasergene and Integrative genomics viewer (IGV)^26^. Genbank accession numbers are listed in supplementary table  4.

### Phylogenetic analysis

All the consensus sequences for HA and NP were compiled into single files and changed to fasta format using online server EMBOSS seqret (EMBL-EBI). The consensus sequences were aligned and analyzed with Clustal Omega (EMBL-EBI). Phylogenetic trees were visualized with Figtree v1.4.4 (http://tree.bio.ed.ac.uk/software/figtree/).

### Virus purification/concentration

Viruses were grown in 10-day old eggs and allantoic fluid was harvested and clarified by centrifugation at 290 x g using a benchtop centrifuge (Eppendorf) at 4 °C for 5 min. The clarified supernatant was concentrated using a 30% sucrose cushion in NTE buffer (100 mM NaCl, 10 mM Tris-HCl, 1 mM ethylenediaminetetraacetic acid (EDTA) at pH 8) by centrifugation in a Beckman L7-65 ultracentrifuge at 87041.1 x g for 2 h at 4 °C using a Beckman SW28 rotor. Supernatant was aspirated and pellets were resuspended in PBS and stored at −80 °C in small aliquots.

### Western blot and protein quantification

Purified viruses were quantified using pierce BCA protein assay kit (Thermo fisher) and 20 ng of protein was mixed with pierce lane marker reducing sample buffer (Thermo fisher). Purified viruses were run on 10% reducing denaturing sodium dodecyl sulfate polyacrylamide gel electrophoresis (SDS-PAGE) (Bio-Rad) and the protein was transferred onto a polyvinylidene difluoride (PVDF) membrane (Bio-Rad) using turbo transfer system (Bio-Rad). The membrane was blocked with 5% (w/v) non-fat milk in PBS containing 0.1% (v/v) Tween 20 (PBST) for 1 h at room temperature on a shaker. The membrane was incubated with KL-BHA-4C10^17^ (1:2000) diluted in 5% non-fat milk in PBST overnight at 4 °C on a shaker. The membrane was washed with PBST three times (5 min incubation each time) on a shaker at room temperature. The membrane was incubated with anti-mouse-HRP (horseradish peroxidase) IgG diluted in 5% milk in PBST (1:2000) for 1 h at room temperature on a shaker. The membrane was washed three times with PBST and developed using Brightstar HCL (ASI). The HRP was killed by incubating the membrane with 1% sodium azide for 15 min on a shaker at room temperature. The membrane is washed overnight with PBST (changed several times) on a shaker. The membrane was incubated with polyclonal antibody against NP (Invitrogen, PA5-81758) diluted in 5% Milk/PBST (1:2000) overnight at 4 °C on a shaker. The membrane was washed three times and incubated with anti-rabbit-HRP diluted in 5% milk/PBST (1:2000) and incubated for 1 h at room temperature on a shaker. The membrane was washed three times and developed with Brightstar HCL. Uncropped version of blots is in supplementary fig. 4.

### Deglycosylation of virion proteins

Purified viruses were quantified using pierce BCA protein assay kit (Thermo fisher) and 20 µg of total protein was treated with PNGase F (New England Biolabs) following manufacturer’s instructions. Briefly, 20 µg purified viruses were mixed with glycoprotein denaturing buffer and denatured by incubating at 100 °C for 10 min. The denatured glycoproteins were cooled on ice and briefly centrifuged using a table top centrifuge (Eppendorf). Glyco Buffer 2, 10% NP-40 and PNGase F was added and the samples were incubated at 37 °C for 1 h. Samples were analyzed by mobility shifts on 12% SDS-PAGE gel (Bio-Rad). The gel was stained with coomassie staining (10% acetic acid (v/v), 40% methanol (v/v), 0.5% R250 brilliant blue R (w/v)) and destained (20% methanol (v/v), 10% acetic acid (v/v)). Uncropped version of gels are in supplementary fig. 3.

### Mass spectrometry/Absolute quantification of HA

Five concentrated influenza viruses (B/Great Lakes/1739/54 WT, B/Hawaii/10/2001 WT, B/Florida/4/2006 MA WT, rM3P67/GL/HN, rM3P67/HI/HN, rM3P67) were first normalized to 50 µg of total protein based on a Bradford assay (Thermo Scientific, according to manufacturer’s instructions) in 150 µL of PBS then 50uL of buffer consisting of 8 M urea (Invitrogen), 200 mM ammonium bicarbonate (Invitrogen), and 2 mM dithiothreitol (DTT) (Invitrogen) was added to achieve a final volume of 200 µL. Samples were incubated in a thermomixer at 60 °C for 30 minutes at 5 × g. Iodoacetamide was added to alkylate free cysteines, and samples were incubated for 45 min in the dark. Samples were then digested with Trypsin Gold (Promega) at a 1:100 (enzyme: protein) ratio and incubated at 37 °C for 16 h. Samples were desalted on a C18 column (mini spin, Nest Group) as per the manufacturer’s protocol. Samples were eluted from these columns with 100 µL 40% ACN/0.1% TFA. Digested samples were dried by vacuum centrifugation and stored at -80 °C until analysis.

Two HA peptides (QLPNLLR and SKPYYTGEHAK) were synthesized containing a C-terminal 13C6,15N2-lysine or 13C6,15N4-arginine residue and quantified by amino acid analysis (Thermo Scientific)^27^. Synthetic peptides were individually diluted from 1 mM to 1fM in 0.5 µg/µL trypsin-digested Bovine Serum Albumin (New England Biolabs) in 0.1% FA and analyzed by a 20-minute mass spectrometry method to determine the range of concentrations that yielded to a linear response with mass spectrometry intensity. Trypsin-digested virus samples were reconstituted in 0.1% formic acid and the synthetic peptides were spiked into the samples to a final concentration of 2.5 µM each, which was within the linear range. Samples were diluted 1:15 and analyzed by a 70-minute mass spectrometry method described below.

All samples were analyzed on an Orbitrap Eclipse mass spectrometry system (Thermo Fisher Scientific) equipped with an Easy nLC 1200 ultra-high pressure liquid chromatography system (Thermo Fisher Scientific) interfaced via a Nanospray Flex nanoelectrospray source. Samples were injected on a C18 reverse phase column (30 cm × 75 μm (ID)) packed with ReprosilPur 1.9 μm particles. Mobile phase A consisted of 0.1% FA, and mobile phase B consisted of 0.1% FA/80% ACN. Peptides were separated by an organic gradient from 5% to 35% mobile phase B over 60 min followed by an increase to 100% B over 10 min at a flow rate of 300 nL/minute. Analytical columns were equilibrated with 3 μL of mobile phase A. All samples were analyzed with the following Data Dependent Acquisition (DDA) settings. An MS1 scan was acquired over a m/z range of 375-1025 in the Orbitrap at 120,000 resolutions (@200 m/z) with a normalized AGC target of 100%, an RF lens setting of 30%, and an instrument-controlled ion injection time. Dynamic exclusion was set to 30 s, with a 10 ppm exclusion width setting. Peptides with charge states 2–6 were selected for MS/MS interrogation using higher energy collisional dissociation (HCD) with a normalized HCD collision energy of 28%, with three seconds of MS/MS scans per cycle. MS/MS spectra were acquired in the Orbitrap at 15,000 resolutions (@200 m/z) with a normalized AGC target of 100%, an RF lens setting of 30% and an instrument-controlled ion injection time and mass range.

To quantify peptide concentrations, precursor ion chromatograms were extracted from the MS1 scans for the synthetic (“heavy”) and endogenous (“light”) HA peptides using Skyline (version 21.2)^28^. Only the 2+ charge states of each precursor were quantified. The peak areas integrated over time was summed for all isotopic peaks of each precursor distribution of each peptide, and the light:heavy peptide ratios were calculated. The final concentration of HA peptide in each sample was calculated by multiplying the light:heavy peptide ratio by the final concentration of the synthetic peptide in the sample (i.e., 2.5 µM). HA content was calculated by averaging the HA peptide concentrations (in molar amounts) for the two peptides and converting HA peptide/protein molarity to HA protein amount (in µg) by multiplying by the molecular weight of HA and the dilution factor used to obtain the 50 µg sample. The % HA content was calculated by dividing the total HA protein amount by the total protein amount measured by Bradford assay.

### Analysis

Statistical analysis was performed using GraphPad Prism (version 9.3.1 (471)). Biorender was used to generate schematics. Adobe illustrator (version 26.0.1) was used to compile the figures.

### Reporting summary

Further information on research design is available in the Nature Research Reporting Summary linked to this article.

## Supplementary information

supplementary file

REPORTING SUMMARY

## Data Availability

The datasets generated and/or analyzed during the current study are available within the paper. GenBank accession numbers and SRA accession numbers are listed in supplementary table 4. The mass spectrometry proteomics data have been deposited to the ProteomeXchange Consortium via the PRIDE^29^ partner repository with the dataset identifier PXD036269.
