# Supplementary material for: Generation of a high yield vaccine backbone for influenza B virus in embryonated chicken eggs
Source: NPJ Vaccines. 2023 Feb 10;8:12. doi: 10.1038/s41541-023-00603-3 (PMC9911942; doi:10.1038/s41541-023-00603-3)
Supplement: Supplementary file 1 — supplementary file [file 41541_2023_603_MOESM1_ESM.pdf]

Supplementary Table 1. Plaque and HA titers for the 31 clonal isolates selected from the co-infection of 15 IBV strains. 31 clonal isolates were selected after two rounds of plaque purification and plaque and HA assay titers are listed. PFU/HAU ratio was calculated to select the top ten strains.

Sample name	*Viral titer*	*HAU*	*PFU/HAU*	*Sample name*	*Viral titer*	*HAU*	*PFU/HAU*
M1P2 1	$1.10x10^9$	128	$8.59 x10^6$	*M3P2 1*	$7.80 x10^8$	512	$1.52 x10^6$
M1P2 3	$7.40x10^8$	256	$2.89 x10^6$	*M3P2 5*	$5.00 x10^8$	256	$1.95 x10^6$
M1P3 1	$6.00 x10^8$	256	$2.34 x10^6$	*M3P5 2*	$5.60 x10^8$	256	$2.19 x10^6$
M1P3 4	$5.40 x10^8$	128	$4.22 x10^6$	*M3P5 5*	$3.60 x10^8$	256	$1.41 x10^6$
M1P4 2	$6.60 x10^8$	128	$5.16 x10^6$	*M3P7 2*	$7.60 x10^8$	256	$2.97 x10^6$
M1P4 3	$5.40 x10^8$	128	$4.22 x10^6$	*M3P8 2*	$4.80 x10^8$	256	$1.88 x10^6$
M1P4 4	$5.20 x10^8$	64	$8.13 x10^6$	*M3P8 4*	$1.16 x10^9$	256	$4.53 x10^6$
M1P4 5	$4.00 x10^8$	256	$1.56 x10^6$	*M3P9 3*	$5.80 x10^8$	256	$2.27 x10^6$
M1P5 5	$6.40 x10^8$	128	$5.00 x10^6$	*M3P9 5*	$7.40 x10^8$	512	$1.45 x10^6$
M2P2 5	$9.40 x10^8$	256	$3.67 x10^6$	*M3P10 1*	$1.34 x10^9$	512	$2.62 x10^6$
M2P3 1	$9.40 x10^8$	128	$7.34 x10^6$	*M2P1 8*	$7.80 x10^8$	256	$3.05 x10^6$
M2P3 3	$1.08 x10^9$	256	$4.22 x10^6$	*M3P6 7*	$1.64 x10^9$	256	$6.41 x10^6$
M3P6 2	$1.28 x10^9$	256	$5.00 x10^6$	*M3P6 8*	$2.60 x10^8$	128	$2.03 x10^6$
M2P4 2	$4.60 x10^8$	128	$3.59 x10^6$	*M3P8 6*	$9.00 x10^8$	512	$1.76 x10^6$
M2P4 3	$7.20 x10^8$	512	$1.41 x10^6$	*M3P8 9*	$4.60 x10^8$	256	$1.80 x10^6$
M2P4 5	$6.00 x10^8$	128	$4.69 x10^6$				

Supplementary Table 2. Quantification of total proteins used for mass spectrometry. Viruses were concentrated using a 30% sucrose cushion and the pellet was resuspended in 2 mL of PBS. Concentration of each virus was measured using Bradford assay and total amount of protein in 2 mL was calculated. Total volume of allantoic fluid used for concentration was noted and used to calculate µg/mL for total protein. HA content (quantified by mass spectrometry) was used to calculate µg of HA per mL of allantoic fluid. (Virus strains used : B/Great Lakes/1739/54, B/Hawaii/10/2001, B/Florida/4/2006 MA, rM3P67/GL/HN, rM3P67/HI/HN, and rM3P67).

Sample	Concentration (µg/µl)	Total protein in 2 mL (µg)	Total allantoic fluid (mL)	Total protein µg/mL	µg of HA /mL
**B/GL/54**	1.6	3200	166	19.3	5.3
**rM3P67/GL/HN**	2.1	4200	163.5	25.7	8.4
**B/HI/2001**	0.7	1400	116.5	12	2.8
**rM3P67/HI/HN**	1.6	3200	112	28.6	6
**B/FL/2006 MA**	2.1	4200	162.5	25.8	8
**rM3P67**	1.8	3600	145	24.8	8

Supplementary table 3. EXTENDED_Genetic characteristics of the top ten clonal isolates. Nucleotide sequences of each segment from the clonal isolates were aligned against the nucleotide sequence of the wild type strains used for co-infection to identify the closest match. Amino acid and nucleotide changes in the coding (numbering is based on ORF) and non-coding (numbering is based on 1st nucleotide) regions are listed in comparison to the wild type sequence, respectively. Silent mutations are italicized. ((NB) indicates mutations in the NB ORF on segment 5/NA, (MA) indicates mouse adapted, and (NS1) indicates mutations in the NS1 ORF on segment 8/NS). (Strain names: B/Florida/4/2006 MA, B/Great Lakes/1739/54, B/Malaysia/2506/2004 MA, B/Michigan/1/71, B/Pennsylvania/5/2007, B/Texas/06/2011, B/Yamagata/16/88, B/Bangladesh/5278/2006).

	**M3P8 4**			**M3P6 7**			**M1P2 1**		
	Strain name	Coding	UTR	Strain name	Coding	UTR	Strain name	Coding	UTR
**PB1**	Penn/07	*T662T*		Penn/07			Penn/07	K741R	
**PB2**	Yamagata /88		G->A (10nt)	Penn/07	*E280E V283V*		Penn/07	*L628L*	
**PA**	Texas/11	K489E		Texas/11	K489E		Texas/11	K489E V553M	
**HA**	Florida/ 06 (MA)			Florida/06 (MA)			Florida/06 (MA)		
**NP**	Texas/11			Michigan/ 71	*L284L*		Michigan/ 71		
**NA**	Michigan/ 71			Yamagata/ 88	P42S T44I (NB)	C->T (1557nt)	Michigan/ 71		
**M**	Texas/11			Texas/11			Texas/11		
**NS**	Penn/07			Penn/07			Malaysia/ 04 (MA)		

	**M2P4 3**			**M3P6 2**			**M2P3 3**		
	Strain name	Coding	UTR	Strain name	Coding	UTR	Strain name	Coding	UTR
**PB1**	Penn/07			Penn/07			Michigan/ 71		A->G (10nt) A->T (12nt)
**PB2**	Penn/07			Penn/07	*E280E V283V*		Yamagata/ 88		G->A (10nt)
**PA**	Texas/11	K489E		Texas/11	K489E		Texas/11	K489E *K298K*	
**HA**	Texas/11			Florida/06 (MA)		C->T (1882nt)	Florida/06 (MA)		
**NP**	Michigan/ 71		Addition of AA at nt 47-48	Michigan/ 71			Michigan/ 71		T->C (1811nt)
**NA**	Michigan/ 71			Yamagata/ 88	P42S T44I (NB)		Michigan/ 71		
**M**	Malaysia/ 04 (MA)	(TAA)stop->(TGA)stop	A->G (1117nt)	Texas/11			Texas/11		
**NS**	Malaysia/ 04 (MA)			Florida/06 (MA)	K70E (NS1)		Malaysia/ 04 (MA)		Deletion of A at nt 45

	**M3P10 1**			**M3P9 5**			**M3P2 1**		
	Strain name	Coding	UTR	Strain name	Coding	UTR	Strain name	Coding	UTR
**PB1**	Penn/07			Penn/07	*T662T*		Penn/07	*Q55Q*	
**PB2**	Bangladesh/06	*L441L*		Yamagata/88		G->A (10nt)	Yamagata/88	*V641V*	G->A (10nt)
**PA**	Texas/11	K489E		Texas/11	K489E		Texas/11	K489E	
**HA**	Florida/06 (MA)			Florida/06 (MA)		C->T (1882nt)	Florida/06 (MA)		
**NP**	Yamagata 88	E535G		Texas/11			Michigan/71		
**NA**	Michigan/71			Michigan/71			Michigan/71		
**M**	Texas/11			Florida/06 (MA)			Texas/11		
**NS**	Malaysia/04 (MA)		Deletion of A at nt 45	Penn/07			Penn/07	*G169G*	

	**M3P8 6**		
	Strain name	Coding	UTR
**PB1**	Penn/07	*T662T*	
**PB2**	Yamagata/88	G632R	G->A (10nt)
**PA**	Texas/11	K489E	
**HA**	Florida/06 (MA)		
**NP**	Texas/11	*A295A*	
**NA**	Michigan/71		C->T (1557nt)
**M**	Texas/11		
**NS**	Penn/07		

Supplementary Table 4. Accession numbers for clonal isolates and WT strains. GenBank and SRA accession numbers for top ten clonal isolates and wild type strains used for co-infection.

STRAIN NAME	GENBANK ACCESSION NUMBER	SRA ACCESSION NUMBER
**M1P2 1**	OQ034397-OQ034404	SRR22846279
**M2P3 3**	OQ034365-OQ034372	SRR22846275
**M3P6 2**	OQ034445-OQ034452	SRR22846264
**M2P4 3**	OQ034349-OQ034356	SRR22846274
**M3P2 1**	OQ034453-OQ034460	SRR22846272
**M3P8 4**	OQ034413-OQ034420	SRR22846278
**M3P9 5**	OQ034437-OQ034444	SRR22846273
**M3P10 1**	OQ034469-OQ034476	SRR22846277
**M3P6 7**	OQ034389-OQ034396	SRR22846265
**M3P8 6**	OQ034341-OQ034348	SRR22846276
**B/GREAT LAKES/1739/54**	OQ034357-OQ034364	SRR22846271
**B/PENNSYLVANIA/5/2007**	OQ034461-OQ034468	SRR22846270
**B/TEXAS/06/2011**	OQ034373-OQ034380	SRR22846266
**B/MICHIGAN/1/71**	OQ034381-OQ034388	SRR22846268
**B/FLORIDA/4/2006 MA**	OQ034421-OQ034428	SRR22846280
**B/MALAYSIA/2506/2004 MA**	OQ034405-OQ034412	SRR22846267
**B/YAMAGATA/16/88**	OQ034429-OQ034436	SRR22846281
**B/BANGLADESH/5278/2006**	OQ034631-OQ034638	SRR22846269

Supplementary Table 5. Segment specific primers used for cloning of IBV segments into pDZ for generating recombinant viruses using infusion cloning.

Name	Sequence
**PB1 forward**	CCGAAGTTGGGGGGGAGCAGAAGCGGAGCCTTTAAGATGAATATAAATCC
**PB1 reverse**	GGCCGCCGGGTTATTAGTAGAAACACGAGCCTTTTTTCATTTTAATCATTTGT
**PB2 forward**	CCGAAGTTGGGGGGGAGCAGAAGCGGAGCGTTTTCAAGATGAC
**PB2 reverse**	GGCCGCCGGGTTATTAGTAGAAACACGAGCATTTTTCACTCAA
**PA forward**	CCGAAGTTGGGGGGGAGCAGAAGCGGTGCGTTTGATTTG
**PA reverse**	GGCCGCCGGGTTATTAGTAGAAACACGTGCATTTTT
**HA forward**	CCGAAGTTGGGGGGGAGCAGAAGCAGAGCATTTTCTAATATCCACAAAATG
**HA reverse**	GGCCGCCGGGTTATTAGTAGTAACAAGAGCATTTTTCAATAACGTTT
**NP forward**	CCGAAGTTGGGGGGGAGCAGAAGCACAGCATTTTCTTGTGAACTTC
**NP reverse**	GGCCGCCGGGTTATTAGTAGAAACAACAGCATTTTTTACATTTATTT
**NA forward**	CCGAAGTTGGGGGGGAGCAGAAGCAGAGCATCTTCTCAAAACTGAAGC
**NA reverse**	GGCCGCCGGGTTATTAGTAGTAACAAGAGCATTTTTCAGAAACA
**M forward**	CCGAAGTTGGGGGGGAGCAGAAGCACGCACTTTCTTAAAATGTCGCTG
**M reverse**	GGCCGCCGGGTTATTAGTAGAAACAACGCACTTTTTCCAGTTTATTT
**NS forward**	CCGAAGTTGGGGGGGAGCAGAAGCAGAGGATTTGTTTAGTCACTGGC
**NS reverse**	GGCCGCCGGGTTATTAGTAGTAACAAGAGGATTTTTATTTTAAATTCACAAGCAC

Supplementary Figure 1. Precursor chromatograms for viruses spiked with heavy HA peptide QLPNLLR. Chromatograms showing precursor intensity over retention time for different viruses (light; shown in red) spiked with labeled HA peptides (heavy; shown in blue). Each chromatogram represents individual virus preparation.

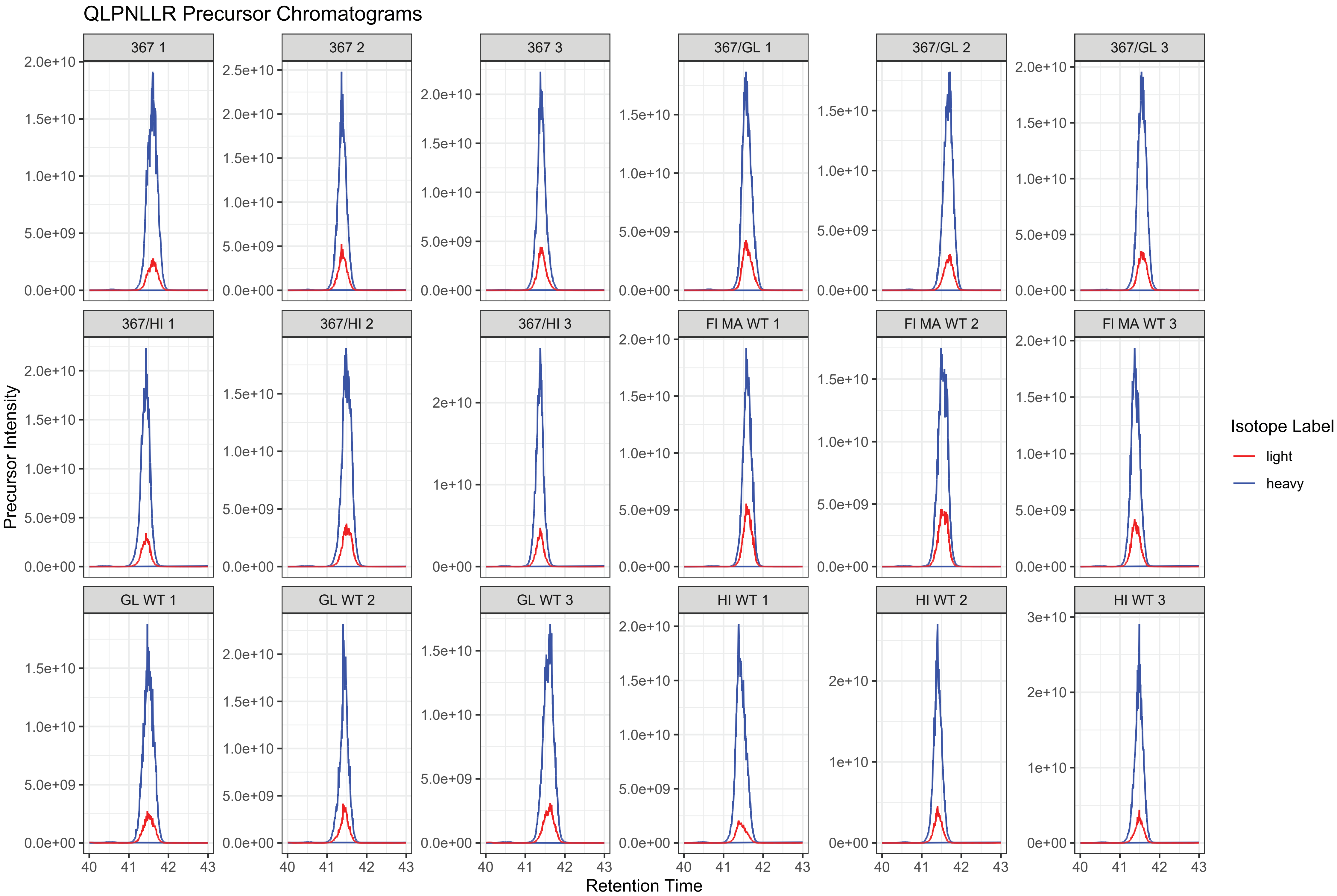

Supplementary figure 1

Supplementary Figure 2. Precursor chromatograms for viruses spiked with heavy HA peptide SKPYYTGEHAK. Chromatograms showing precursor intensity over retention time for different viruses (light; shown in red) spiked with labeled HA peptides (heavy; shown in blue). Each chromatogram represents individual virus preparation.

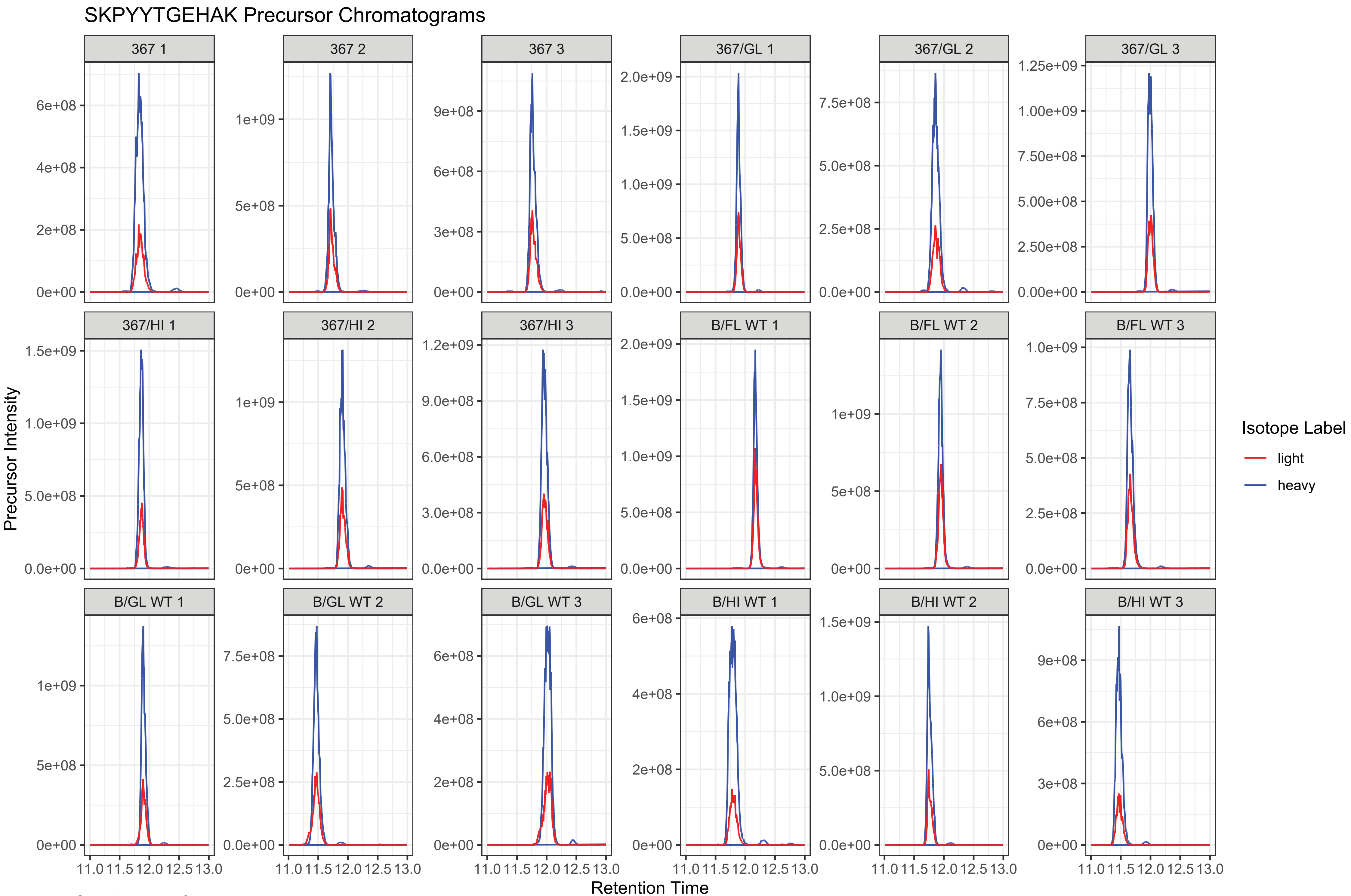

Supplementary figure 2

a

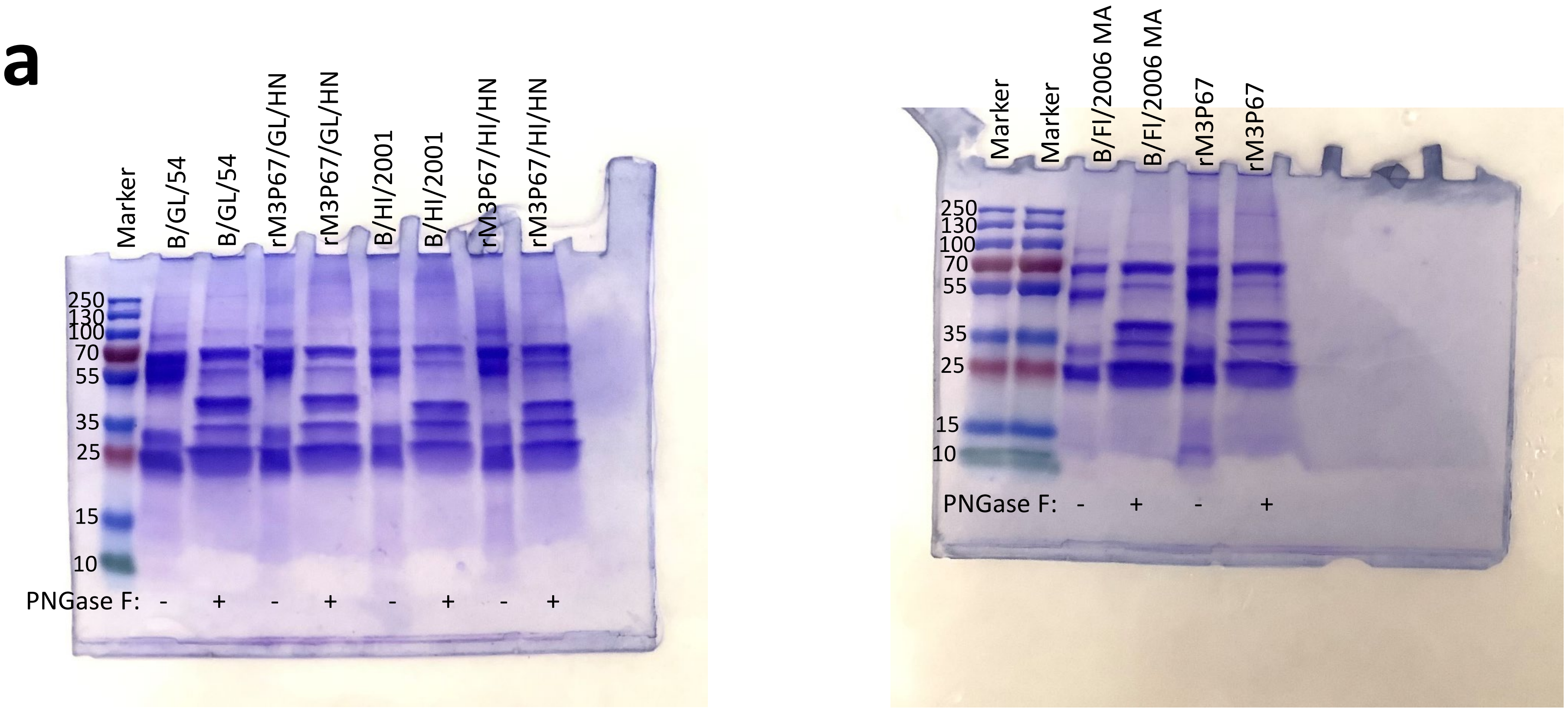

Supplementary figure 3: Characterization of HA content in the reassortant and WT viruses. Un-cropped/edited version of gels from figure 6a.

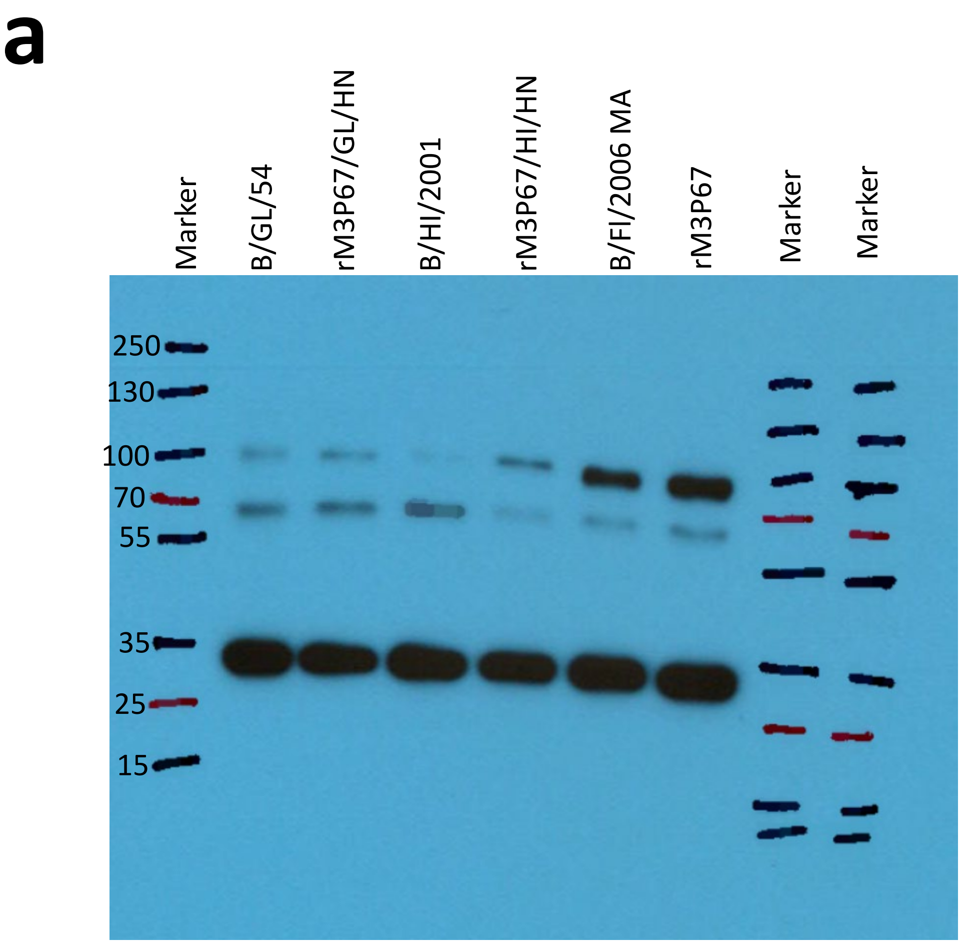

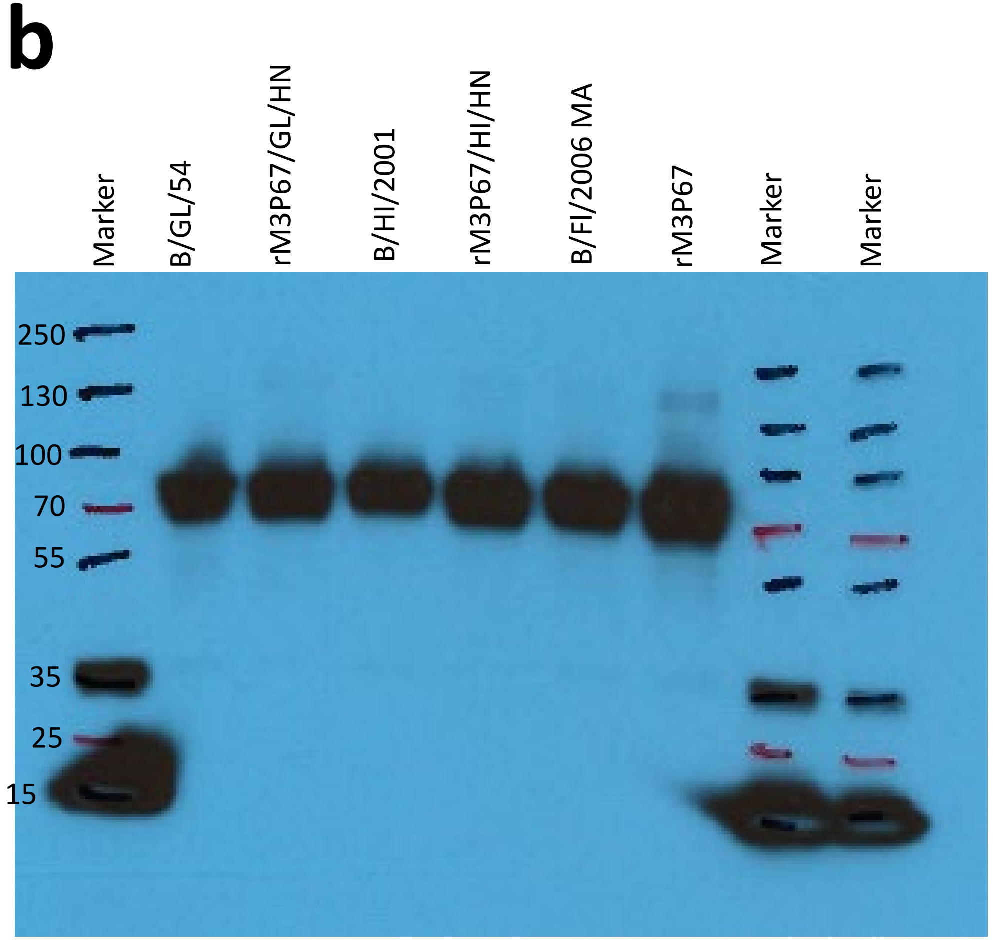

Supplementary figure 4: Characterization of HA content in the reassortant and WT viruses. Un-cropped/edited version of blots from figure 6b.
